# Complex networks approach to study comorbidities in patients with unruptured intracranial aneurysms

**DOI:** 10.1038/s41598-024-59919-2

**Published:** 2024-04-22

**Authors:** Juri Kivelev, Ilkka Saarenpää, Antti Karlsson, Paride Crisafulli, Federico Musciotto, Jyrki Piilo, Rosario N. Mantegna

**Affiliations:** 1 Department of Neurosurgery, Turku University Hospital, Turku, Finland; 2 Auria Biobank , University of Turku, Turku, Finland; 3 Dipartimento di Fisica e Chimica, Università degli Studi di Palermo, Palermo, Italy; 4 Instituto de Fısica Interdisciplinar y Sistemas Complejos IFISC (CSIC-UIB), 07122 Palma de Mallorca, Spain; 5 Department of Physics and Astronomy, University of Turku, Turku, Finland; 6 Complexity Science Hub, Vienna, Austria

**Keywords:** Computational neuroscience, Mathematics and computing

## Abstract

The role of complex network analysis in patients with diagnosis of unruptured intracranial aneurysm is unexplored. The objective of this study is to assess the applicability of this methodology in aneurysm patients. We retrospectively analyze comprehensive unbiased local digital data of a large number of patients treated for any reason between January 2004 and July 2019. We apply an age-cohort approach to a total of 628,831 patients and construct the diagnostic history of each patient—and include the information how old the patient was when diagnosed for the first time with each diagnosis coded according to International Classification of Diseases. For each cohort of age within a 10 year interval and for each gender, we construct a statistically validated comorbidity network and focused on crucial comorbidity links that the aneurysm code has to other disease codes within the whole network. For all cohorts of different age and gender, the analysis shows that 267 diagnose codes have nearest neighbour statistically validated links to unruptured aneurysm ICD code. Among the 267 comorbidities, 204 (76%) were found in patients aged from 40 to 69-years old. Patterns of connectivity with aneurysms were found for smoking, hypertension, chronic obstructive pulmonary disease, dyslipidemia, and mood disorders. A few uncommon connections are also detected in cohorts of female patients. Our study explored the applicability of network analysis and statistical validation in aneurysm observational study.

## Introduction

Complex network theory has shown its applicability in biomedical research providing new insights in understanding nature and mechanisms of a number of diseases^1–3^. More than a decade ago, Goh et al. presented the first report on applicability of network theory in analyzing the links of genetic disorders resulting in “diseasome”, the combined set of all known disorder/disease gene associations^4^. Furthermore, Hidalgo et al. proposed a phenotypic disease network analysis which showed the potential in unraveling the disease progression when considering the role of comorbidities^5^. The role of complex network analysis of comorbidities in early diagnosing of unruptured intracranial aneurysm (UIA) is worth to be investigated. In modern practice, most of UIA are found incidentally on magnetic resonance imaging or computerized tomography. Some of incidental UIA are treated prophylactically aiming at preventing subarachnoid hemorrhage, a devastating intracranial catastrophe, which carries approximately 30–40 % mortality risk and causes neurological disability in significant part of the survivors^6,7^. Recommendations regarding indications for screening non-familial UIA in general population are still lacking scientific evidence and vary according to institutional preferences. Nowadays, medical digital data and their non-censored computerized processing have reinforced the methodological armamentarium of investigators allowing re-assessment of previous clinical guidelines in management of UIA. To benefit the automated data collection and network statistics in clinical research, we established a digital big data-type dataset of all available codes of diseases coded within International Codes of Disease (later - ICD) 10 classification encountered in all consecutive patients treated for any reason in the wellbeing servicies county of Southwest Finland. Subsequently, we performed a network analysis of ICD codes of comorbidities in UIA patients. The objective of this study is to present the applicability and effectiveness of a complex network investigation in assessing the role of the comorbidities in screening and management of patients with unruptured intracranial aneurysms before haemorrhage.

## Results

We process medical records of a dataset including 628,831 individuals treated to any reason within 15 years period. Having this big data-type sample of information we apply complex network analysis in studying the role of comorbidities in patients carrying UIA. Specifically, we extract comorbidity networks from the diagnoses history of patients of the wellbeing services county of southwest Finland. Diagnoses span a period of 15 years and 7 months providing enough information for the study of comorbidity of unrupted aneurysms in groups of patients of different age and gender. Diagnoses are coded electronically in terms of the international statistical classification of diseases (ICD) of the World Health Organization. Diagnoses are highly heterogeneous in their occurrence in the population, some diseases are highly frequent, and others occur only rarely. For this reason, we use a complex network methodology^8^ that is robust with respect to the occurrence of the elements of interest (in the present case the number of diagnoses of a given disease). The complex networks subgraphs obtained with this methodology are called statistically validated networks^8^. We briefly describe this methodology in the methods section. In the following sections, we describe the database investigated and the statistically validated networks of ICD codes obtained for groups of patients of given gender and age class. Our focus will be on the so-called “ego network" of disease I67.1, i.e., of the disease classified as “Cerebral aneurysm, nonruptured". An “ego network" is a subgraph of a network consisting of the node of interest (in our case I67.1), and all nodes directly connected with it. The validation method for the links in networks used in our clinical study, allows to overcome the problem of heterogeneity in terms of prevalence and patient-wise number of diagnoses. By starting from the empirically observed bipartite disease-patient network the link-validation procedure allows extraction of the most informative structure of the comorbidity network from the general data sample eliminating the bias arising due to diseases’ heterogeneity. Subsequently, we could focus on the specific part of the validated network, i.e., UIA ego-network, and managed to highlight the set of links related to diagnosis of UIA. After applying age-cohort approach we can comprehensively overview the comorbidity sets and their connectivity patterns for each age-decade in both genders. From a practical point, this brings an additional prospective in analyzing aneurysm epidemiological phenotype-based data.

We show that “ego networks" of I67.1 are informative with respect to: (i) prominent comorbidities signaling higher risk of presence of unrupted aneurysms in patients suffering them, (ii) role of gender and age class in I67.1 comorbidities, and (iii) additional indicators of increased potential risk of unruptured aneurysm. In the next sections, we present and discuss data and ego networks of I67.1 extracted from statistically validated networks. The method used to obtain statistically validated networks is described in the method section.

## Data

### Availability of data materials

The original data cannot be shared due to being proprietary data of Auria Clinical Informatics which operates in connection with the wellbeing services county of Southwest Finland. The study was approved by the Institutional Review board of Turku University Hospital (license number T152/2017). Informed consent was waived due to the retrospective design of the study according to Finnish legislation on secondary use of health data.

### Data preprocessing

This is a population-based longitudinal retrospective study. Our data consisted of the time period between the 1st of January 2004 and 31st of July 2019 and included 628,831 individuals. We divide the patients in groups by gender and age. Since the data does not cover all the life span of the patients, there is no unique way how to construct the age cohorts. For the present study, we have chosen the following procedure. We start considering first the diagnoses history of each patient and record how old the patient was when diagnosed for the first time with each of his/her ICD codes (note: we use ICD code level XX.X). After this, we construct the age-gender cohorts for the duration of 10 years—0–9 years old, 10–19 years old and so on—and keep for each patient the entire history of his/her diagnoses. For example: if patient is diagnosed for the first time with ICD code 1 when being 56 years old and has a first-time diagnoses with ICD code 2 when having age 63, then the first data point contributes to the 50–59 cohort and both data points contribute to the 60–69 age cohort. In this way, we are able to track in comprehensive way the long-term diagnoses history. Data was processed and the statistically validated comorbidity network of ICD codes implemented utilizing a software that we developed for this purpose. The software can be downloaded at the repository https://github.com/complexParide/svalnet.

### Ego network

**Figure 1:**
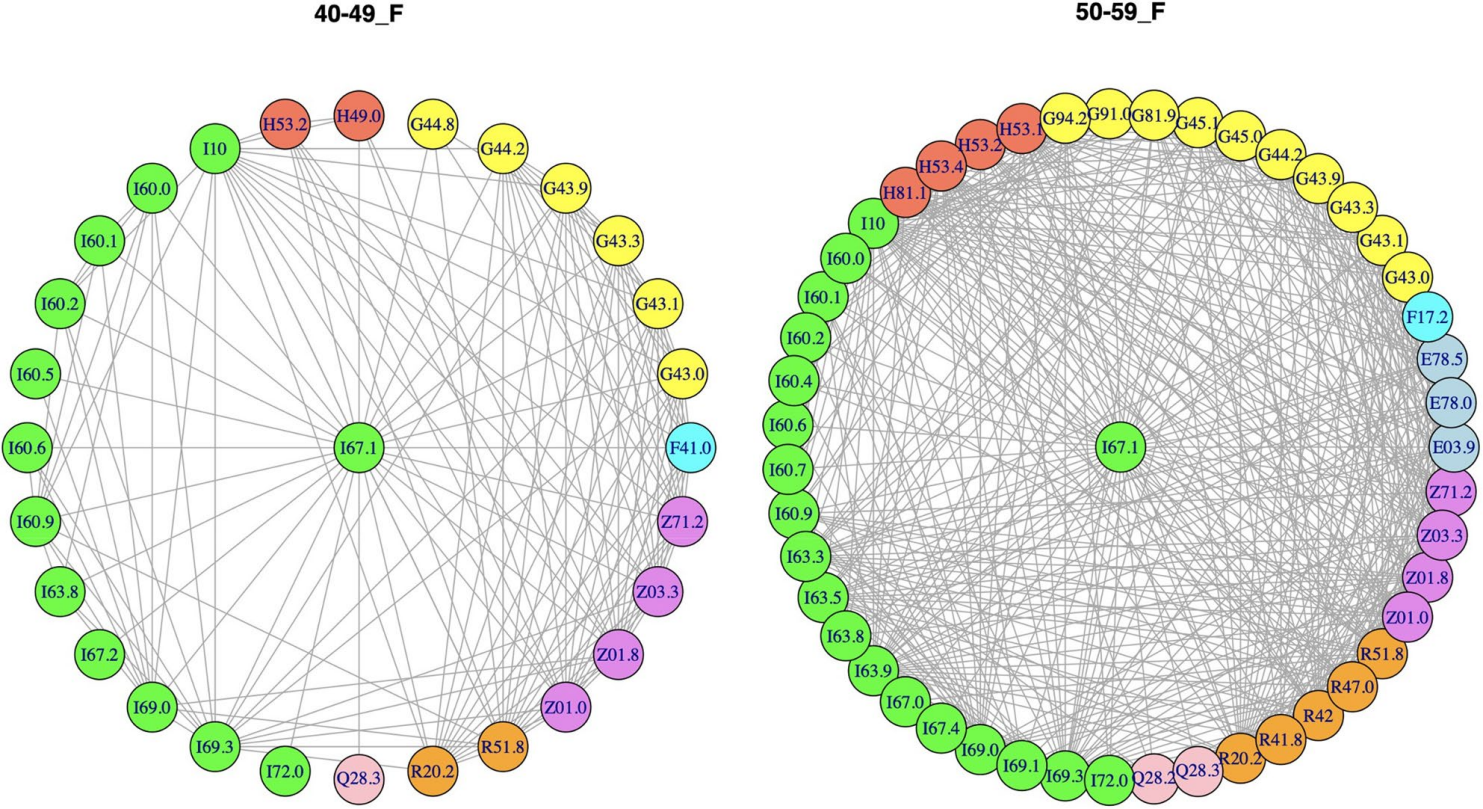
Ego networks of ICD67.7 UIA for women of age class from 40 to 49 (left panel) and from 50 to 59 (right panel). Each node is labeled with its ICD code. Different colors are used for different categories of ICD codes. Specifically, E: endocrine, nutritional and metabolic diseases, light blue; F: mental and behavioural disorders, cyan; G: diseases of the nervous system, yellow; H: diseases of the eye and adnexa (H00–H59) and diseases of the ear and mastoid process (H60–H95) , coral; I: diseases of the circulatory system, green; Q: congenital malformations, deformations and chromosomal abnormalities, pink; R: symptoms, signs and abnormal clinical and laboratory findings, not elsewhere classified, orange; Z: factors influencing health status and contact with health services, violet.

Having thereby constructed the statistically validated comorbidity network of all ICD codes, we consider the ICD code I67.1, meaning UIA, under detailed scrutiny. Specifically, we observe the “ego-network” of I67.1. In networks for UIA (ICD code I67.1), we found several groups of diseases linked to UIA through statistically validated links. By stratifying over the genders, the analysis selects in total 77 distinct ICD codes which are connected by statistically validated links to UIA and are dependent on the age and gender of the diagnosed. Figure 1 displays example networks for females for age groups 40–49 and 50–59. Statistically validated networks for all groups of age and gender and their description are given in the Supplemental Information. In their own networks men have 42 distinct ICD nodes and women have 73 ICD nodes. For a summary of their stratification with respect to the different categories of ICD code, age and gender, see Table 1. For the complete list of ICD presence in the “ego networks" of ICD67.1 for all investigated groups of patients see Table S1A, S1B and S2 of the Supplemental Information. Headaches (R51.80) were linked to UIA code in all patients regardless age (with the exception of 80-XX Women) and gender. This obviously corresponds to the real-life situation when a brain imaging is commonly performed on ambulatory bases due to patient’s complaint of headache. As a result, UIA is detected frequently as an incidental finding. Prominent co-morbidities with UIA are diseases of the circulatory system. A link to hypertension (ICD code I10) exists in all patients with age between 40 and 79 years regardless their gender. An ischemic and hemorrhagic stroke with or without confirmed atherosclerosis (ICD code level I6x.xx) and brain vessel malformations (ICD code level Q2X.XX) are the most typical connections to UIA in all patients. These links are almost exclusive in men younger than 40 years and are the larger fraction of links in women. The codes of stroke also included the condition after the brain aneurysm rupture (I60.XX). Such link was typical for patients with multiple aneurysms, when unruptured one (I67.1) was detected after or before the rupture of another one in the same patient. Smoking (ICD codes Z72.0 and F17.2) is associated with UIA diagnosis in both genders in persons withe age from 40 to 79 years. Dyslipidemia (ICD codes E78.X) has connectivity to UIA in all women from 50 to 79 years, whereas the respective figure was observed in men only from 60 to 69 years of age. Moreover, a link to chronic obstructive pulmonary disease (COPD) coded with J44.8 was found only in women aged 60–69 and 70–79 years-old whereas none of the pulmonary disease code could be connected to men with UIA. Mood disorders showed connectivity with UIA in women aged from 40 to 49 years (panic disorder—ICD code F41.0) and in men aged from 50 to 59 years (depression—ICD code F32.9).

**Table 1:** Table with numbers of nodes in I67.1 ego-network of statistically validated networks.

Age	E00–E99		F00–F99		G00–G99		H00–H99		I00–I99		J00–J99		M00–M99		Q00–Q99		R00–R99		Z00–Z99		Total	
	M	W	M	W	M	W	M	W	M	W	M	W	M	W	M	W	M	W	M	W	M	W
30–39	–	–	–	–	–	1 (10)	–	–	6 (85.7)	6 (60)	–	–	–	–	–	1 (10)	1 (14.3)	1 (10)	–	1 (10)	7	10
40–49	–	–	1 (5.3)	1 (3.6)	2 (10.5)	6 (21.4)	–	2 (7.1)	14 (73.7)	12 (42.9)	–	–	–	–	–	1 (3.6)	1 (5.3)	2 (7.1)	1 (5.3)	4 (14.3)	19	28
50–59	–	3 (6.4)	1 (3.4)	1 (2.1)	3 (10.3)	10 (21.3)	2 (6.9)	4 (8.5)	15 (51.7)	18 (38.3)	–	–	–	–	1 (3.4)	2 (4.3)	1 (3.4)	5 (10.6)	6 (20.7)	4 (8.5)	29	47
60–69	2 (7.1)	3 (5.7)	1 (3.6)	–	3 (10.7)	9 (17.0)	–	2 (3.8)	14 (50)	21 (39.6)	–	1 (1.9)	–	–	1 (3.6)	3 (5.7)	4 (14.3)	7 (13.2)	3 (10.7)	7 (13.2)	28	53
70–79	–	2 (5.9)	–	1 (2.9)	–	4 (11.8)	–	–	7 (63.6)	13 (38.2)	–	2 (5.9)	–	1 (2.9)	1 (9.1)	3 (8.8)	2 (18.2)	3 (8.8)	1 (9.1)	5 (14.7)	11	34
80–XX	–	–	–	–	–	–	–	1 (100)	–	–	–	–	–	–	–	–	–	–	–	–	–	1

Percent values are given in parenthesis and are obtained by considering the total number in each row for men (M) and women (W) separately.

The age cohorts from 60 to 69 and 70 to 79 years old of women present a link to Q61.2 Polycystic kidney, autosomal dominant (see supplemental information Figures S4 and S5 and Table S1B). This observation confirms the conclusion that some monoigenic conditions, such as autosomal dominant polycystic kidney disease are associated with UIA^9,10^.

In addition to the verification of comorbidity well known in literature, our method highlights a number of less documented comorbidities that could be used as alerts to be used to trigger dedicated analyses. For example, vision (H49.0 and H53.X) and hearing (H81.1, H93.1 and R42) disorders are systematically detected in women and are also present in men in the cohort from 50 to 79 years (see Tables S1A and S2 of the supplemental information). Some encountered comorbidities observed in women were mostly related to aneurysms and/or the represented indications for brain imaging and included dysphasia/aphasia (R47.0) and paraesthesia of skin (R20.2).

## Discussion

Our findings show the applicability of network analysis methodology to population-based study of co-morbidities in patients harboring unruptured intracranial aneurysms. Statistically validated networks matched to patients’ age and gender elucidated certain groups of diseases detected in UIA carriers. Of note, in real-life conditions more than 90% of UIA are found incidentally on MRI and/or CT imaging. Thus, the observed links in our networks do not confirm actual clinical causality of respective co-morbidities and UIA, but indeed show their co-existence on populational level from the statistical point of view. In this respect, we can subdivide the revealed co-morbidities from clinical point of view to three general types that we label A, B and C. Specifically, the three types are defined as follows: (A) diseases generally accepted to occur in UIA carriers, (B) diseases representing clinical situations when the indication for neuroimaging is related to diseases of central nervous system and showing UIA as an incidental finding, and (C) previously unproven connection to UIA. We think the data in group C is of utmost interest implying the need for further clinical research to discover the actual role of these pathological conditions in developing an UIA. As an example, one can appreciate the distribution of aforementioned groups in both genders showed in Tables S1A, S1B and S2 in the supplemental information. Co-morbidities of group C are primarily detected in women. Specifically, they are diseases like Streptococcal pharyngitis (ICD J02.0)^11,12^, chronic obstructive pulmonary disease (ICD J44.8)^13^, hypothyroidism (ICD E03.9)^14,15^ and panic disorder (ICD F41.0). In men, group C comorbidity is limited to depression (ICD F32.9).

Since the prevalence of UIA is estimated to be 3%, accounting up to 162,000 cases in Finland and 6,5 million cases in the USA, new data on potentially causative comorbidities in this patients may carry practical value^6,16,17^. Systematic meta-analysis studies^16^ have shown that prevalence ratios in Finland, Japan and US are similar. According to recent a Finnish study analyzing the nationwide Cause of Death and Hospital Discharge Registers from 1998 to 2017, rupture of the aneurysm and subarachnoid hemorrhage was the most common type of stroke discovered that aneurysmal SAH represented the 18th most common cause of death in all middle-aged persons in Finland^18^. These data advocate the development of efficient algorithms for general population screening and reasonable management of the incidental UIA, especially in working-aged individuals. In this sense, our study on network analysis of co-morbidities carries a potential for formulating individualized medically and socioeconomically adjusted UIA management solutions in real-life clinical settings. In practical medicine, physicians frequently face the question whether screening of healthy individuals in terms of non-familial UIA diagnosing is reasonable or not. To date, the widely accepted concept of what are the risk groups suitable for the screening of non-familial UIA has not been established. In the literature, the risk of developing of UIA is reported only among persons with certain connective-tissue disorders (e.g., the Ehlers–Danlos syndrome), and among persons with polycystic kidney disease^19,20^. However, due to rareness of aforementioned entities, their role in epidemiology of UIA within the general population is still debated^10^. In our case, our data do not contain genetics information about patients and therefore we cannot perform investigations on the genetic profile of them. Further, we list several potential benefits of comorbidity networks in unruptured aneurysm patients, which may open new insights for further clinical investigations.

Concerning benefits and limitations, first, networks address the set of diseases most probably related to UIA which may have a role in developing of UIA before the moment of diagnosing. Nowadays, physicians tend to benefit increasingly from non-invasive diagnostic solutions and guidelines based on computerized analysis of in-hospital medical registries. Indeed, in the real-life situation, an ambulatory access to biological material from each individual patient for mapping the genomic or any other omics-based personalized risk stratification in terms of UIA screening seems to be cumbersome and even irrational. Thus, automatized identification of certain sets of co-morbidities from medical registers or other available digital data sources may indicate the risk of developing UIA, thus facilitating the decision-making. Second, uncovered by our analysis age-matched set of co-morbidities associated with UIA may support the physician in assessment of potential risks of active treatment, especially in cases when the patient’s previous health status is not thoroughly investigated. For example, the discovered connectivity between chronic obstructive pulmonary diseases and UIA in women older than 60 years can direct a diagnostic workout toward identifying hidden subclinical forms of COPD in non-smoking aneurysm carries in the respective age. The previous paradigm of age limitation (not older than 65 years) in selection cases for an active treatment of UIA currently is shifting toward higher age limits and is more dependent on the patient’s co-morbidities, functional status, and local life-expectancy rates^21^. The sets of co-morbidities discovered in our age and gender stratified networks may help in the prediction of health status and phenotypical changes in UIA patients even in the long run, thus helping to optimize a timing of active treatment. This is especially relevant in cases, when UIA is diagnosed in young or middle age and considered for conservative management, but later follow-up advocates an active treatment of the aneurysm for some reason. In such cases, treatment safety may be secured by early and focused prevention of diseases and conditions which tend to accumulate with aging specifically in UIA patients.

Despite of the large sample size, our network analysis was performed on a homogeneous population from the single-center area. Furthermore, the availability of neuroimaging, the accuracy of disease coding by healthcare providers and the degree of digitalization of patient charts may be a limitation in generalization of our networks. The conceptual disadvantage of UIA network may be faced in interpretation of the connectivity of the links to the diagnosis of interests and between each other. Although the likelihood of random connectivity between the nodes in validated networks is fairly negligible the existed links still do not necessary explain their causality. In this context, discovered ICD codes should be estimated as a phenotypical characterization of the selected patient group with UIA which has a certain set of diseases prone to appear with stratified connectivity patterns.

Statistically validated networks processed by analyzing an unbiased dataset of 628,831 consecutive patients demonstrated high diversity of comorbidities in patients with unruptured intracranial aneurysms. Regardless heterogeneity of primary database, we addressed age and gender stratified clusters of comorbidities which had respective connectivity patterns. Regardless methodological limitations, the existed data may be used for further research in prediction of the dynamics of health status in patients with newly diagnosed unruptured aneurysms in the long run.

## Methods

**Figure 2:**
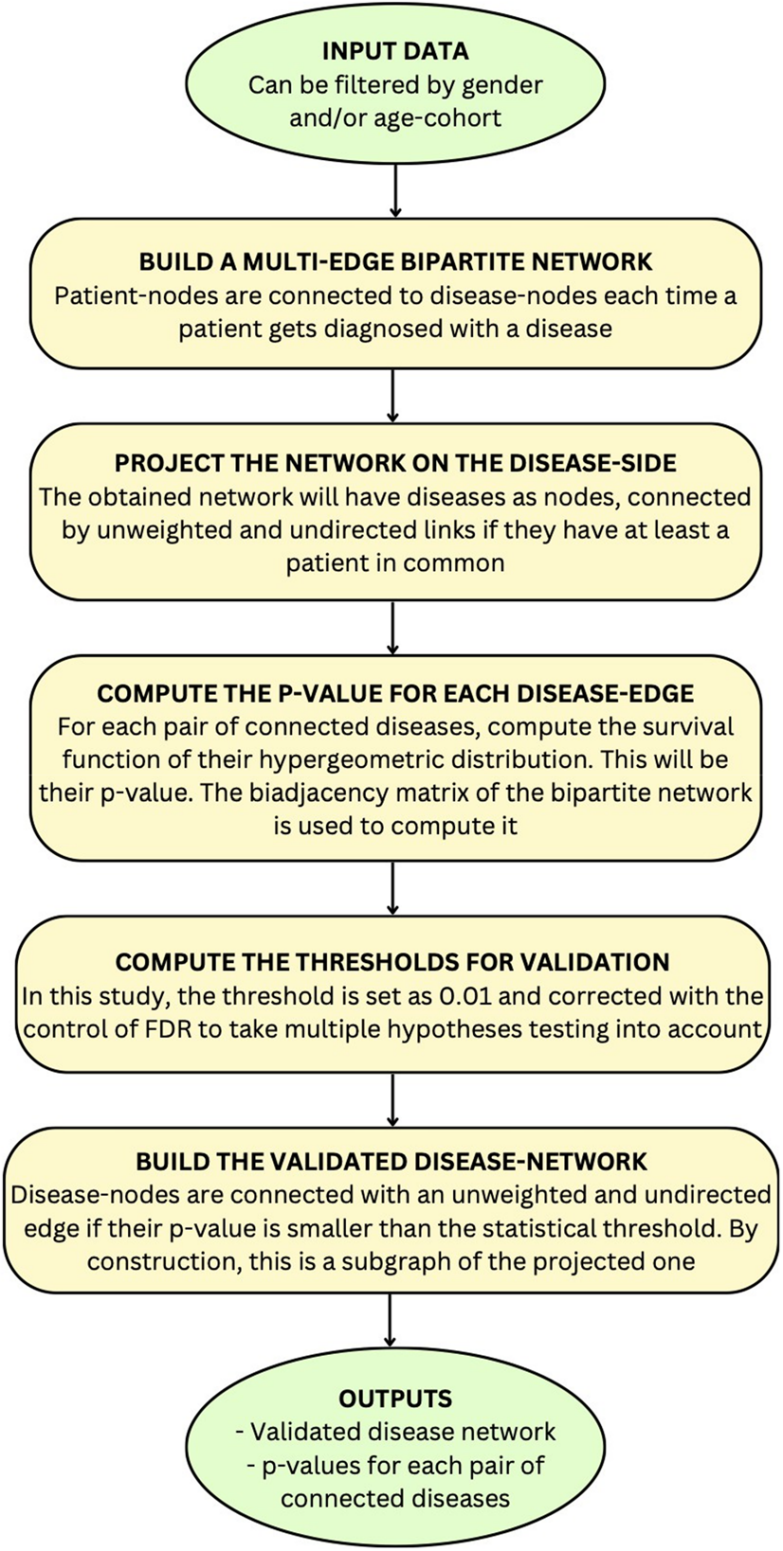
Scheme of the validation procedure of the disease comorbidity network. The different steps are detailed in the section about “Methods”.

In this study, all methods were performed in accordance with the relevant guidelines and regulations including the Declaration of Helsinki. Staring from medical records, for groups of patients characterized by their age and gender we obtain statistically validated networks of comorbidity of diseases. From these statistically validated networks we extract the Ego networks related to the unruptured aneurysm (I67.1). The different steps of our method are shown in Fig. 2.

### Network concepts

A network consists of a set of N nodes where pairs of nodes are connected with links. The degree of a given node *i*, *k*_*i*_, quantifies to how many other nodes the node *i* is connected to. Having the knowledge of the degree of all the nodes in the network via degree sequence *k*_*i*_, one can construct, e.g., the degree distribution *P*(*k*). This gives the probability that a node has degree equal to *k*, when picked up uniformly random from the network. In a bipartite network, we have two set of nodes—A and B—and the links that join nodes from the set A to the set B. From the bipartite network, it is possible to construct a one-mode network for the nodes in the set A (B) by projecting out the nodes of the set B (A). For our current purpose, the set A consists of ICD codes of medical diagnoses and the set B consists of patients. There is a link between the ICD code and the patient if the patient has been diagnosed with this ICD code. By using our database and projecting now on the side of the ICD codes, we obtain an empirical network of diagnoses where there is a link between two ICD codes if there exists at least one patient who has been diagnosed with both codes.

### Statistical validation of networks

The empirical data used to construct networks might present spurious co-occurrence and—perhaps even more importantly—the data features heterogeneity. In our case, this means that the prevalence of ICD codes can vary several orders of magnitudes depending on the code. In other words, some of the diseases are very rare affecting only a few patients while some other diseases are very common, most of the population/cohort having been diagnosed the corresponding ICD code at some point of their life.

### Null hypothesis

The statistically validated network (SVN) method^8^ is based on choosing an appropriate null hypothesis which fully accounts for the heterogenous properties of the system and network under the study. As a starting point, the method is based on constructing the bipartite network being studied. In the current case, we have a bipartite network of ICD codes and patients, where the nodes from the two sets are linked as described above. Now, the null hypothesis is based on randomizing this bipartite network by maintaining the empirically observed degree of nodes of set A and set B. In the corresponding randomized network, the number of ICD codes each patient had remains the same, and the prevalence of each ICD code also remains the same. However, for each patient his/her ICD codes are chosen randomly, and for each ICD code—in turn—the patients are chosen randomly. From the point of view of the bipartite network, this corresponds to random rewiring of the network where the degree of each node is however kept fixed and the degrees still corresponds to the original non-randomized network.

### P-value

With the null-hypothesis described above, it now becomes possible to calculate the *p*-value associated with the number of co-occurrences observed for each pair of the ICD codes co-occurring among patients. Suppose that *N*_*i*_ (*N*_*j*_) is the empirically observed number of the patients having been diagnosed with the ICD code *i* (*j*). *N*, in turn, indicates the total number of the patientsX in the whole data set and *X* notes the number of co-occurrences of the diagnoses with both the ICD codes *i* and *j*. The probability that the fully randomized network—corresponding to the null hypothesis—would present a number *X* of co-occurrences can now be calculated from by the hypergeometric probability distribution as

1 $$H(X|N,N_i,N_j)=\frac{{N_i \choose X} {N-N_i \choose N_j-X}}{{N \choose N_j}}.$$

Consider now that *N*_*i, j*_ is the actual number of co-occurrences observed in the original network. Subsequently, the *p*-value for having observed the number *N*_*i, j*_, or more, co-occurrences in the randomized network is given by

2 $$p(N_{i,j}) =1- \sum_{X=0}^{N_{i,j}-1} H(X|N,N_i,N_j).$$

### Statistical validation: multiple hypothesis testing

Most often, the empirical constructed networks are very large having a large number of nodes and links. With the above- mentioned validation method, we actually statistically select links rejecting a null hypothesis between all possible pairs of the nodes. Therefore, also the number of statistical tests becomes large. Thus, when performing a statistical test for a specific pair of nodes—to minimize the number of false positive—we have to use methods suitable for a multiple hypothesis test procedure. For this purpose, we use the control of the false discovery rate (FDR) procedure^22^. We apply the control of the FDR as follows, we first order all calculated *p*-values corresponding to the validation of each test/link, to an increasing order. The

actual threshold value increases linearly with the location and the corresponding index of the *p*-value/test in their ordered set. When we reach the point where the threshold is no more satisfied, i.e., *p*_*n*_ > *nα*, where *α* is the used fixed threshold value (in our case *α* = 0*.*01). The multiple hypothesis test procedure with the control of FDR therefore detects pairwise rejection of the null hypothesis of random matching of diseases highlighting comorbidity relations that are statistically validated by taking into account potential familywise errors.

### Supplementary Information

Supplementary Information.

## References

[1] Barabási A-L (2007). Network Medicine—From Obesity to the “Diseasome”. N. Engl. J. Med..

[2] Goh K-I, Choi I-G (2012). Exploring the human diseasome: The human disease network. Brief. Funct. Genomics.

[3] Jiang Y, Ma S, Shia B-C, Lee T-S (2018). An epidemiological human disease network derived from disease co-occurrence in Taiwan. Sci. Rep..

[4] Goh K-I (2007). The human disease network. Proc. Natl. Acad. Sci..

[5] Hidalgo CA, Blumm N, Barabási A-L, Christakis NA (2009). A dynamic network approach for the study of human phenotypes. PLoS Comput. Biol..

[6] Nieuwkamp DJ (2009). Changes in case fatality of aneurysmal subarachnoid haemorrhage over time, according to age, sex, and region: a meta-analysis. Lancet Neurol..

[7] Brown RD, Broderick JP (2014). Unruptured intracranial aneurysms: Epidemiology, natural history, management options, and familial screening. Lancet Neurol..

[8] Tumminello M, Micciche S, Lillo F, Piilo J, Mantegna RN (2011). Statistically validated networks in bipartite complex systems. PloS one.

[9] Zhou Z (2017). Is regular screening for intracranial aneurysm necessary in patients with autosomal dominant polycystic kidney disease? A systematic review and meta-analysis. Cerebrovasc. Dis..

[10] Bakker MK, Ruigrok YM (2021). Genetics of intracranial aneurysms. Stroke.

[11] Pyysalo MJ (2016). Bacterial DNA findings in ruptured and unruptured intracranial aneurysms. Acta Odontol. Scand..

[12] Avallone SV, Levy AS, Starke RM (2021). A rare case of *Streptococcus anginosus* infectious intracranial aneurysm: Proper management of a poor prognosis. Surg. Neurol. Int..

[13] Stroh N (2023). Machine learning based outcome prediction of microsurgically treated unruptured intracranial aneurysms. Sci. Rep..

[14] Atchaneeyasakul K (2017). Association of hypothyroidism with unruptured cerebral aneurysms: A case–control study. J. Neurosurg..

[15] Park H (2023). Is thyroid dysfunction associated with unruptured intracranial aneurysms? A population-based, nested case–control study from Korea. Thyroid.

[16] Vlak MH, Algra A, Brandenburg R, Rinkel GJ (2011). Prevalence of unruptured intracranial aneurysms, with emphasis on sex, age, comorbidity, country, and time period: A systematic review and meta-analysis. Lancet Neurol..

[17] Cras TY (2020). Determinants of the presence and size of intracranial aneurysms in the general population: The Rotterdam study. Stroke.

[18] Asikainen A, Korja M, Kaprio J, Rautalin I (2023). Case fatality of aneurysmal subarachnoid hemorrhage varies by geographic region within Finland: A nationwide register-based study. Neurology.

[19] Bor ASE, Koffijberg H, Wermer MJ, Rinkel GJ (2010). Optimal screening strategy for familial intracranial aneurysms: A cost-effectiveness analysis. Neurology.

[20] Broderick JP (2009). Greater rupture risk for familial as compared to sporadic unruptured intracranial aneurysms. Stroke.

[21] Lee SH (2021). Clinical outcomes of clipping and coiling in elderly patients with unruptured cerebral aneurysms: a national cohort study in korea. J. Korean Med. Sci..

[22] Benjamini Y, Hochberg Y (1995). Controlling the false discovery rate: A practical and powerful approach to multiple testing. J. R. Stat. Soc. Ser. B (Methodol.).

