# Supplementary material for: Complex networks approach to study comorbidities in patients with unruptured intracranial aneurysms
Source: Sci Rep. 2024 Apr 22;14:9175. doi: 10.1038/s41598-024-59919-2 (PMC11035559; doi:10.1038/s41598-024-59919-2)
Supplement: Supplementary file 1 — Supplementary Information. [file 41598_2024_59919_MOESM1_ESM.docx]

**Supplemental information**

*Complex networks approach to study comorbidities in patients with unruptured intracranial aneurysms*

Authors

Juri Kivelev^1^ , Ilkka Saarenpää^1^ , Antti Karlsson^2^ , Paride Crisafulli^3,4^, Federico Musciotto^3^ , Jyrki Piilo^5^ , Rosario N. Mantegna^3,6^

^1^ – Department of Neurosurgery, Turku University Hospital, Turku, Finland

^2^ – Auria Biobank, Turku University Hospital, University of Turku, Finland

^3^ – Dipartimento di Fisica e Chimica Emilio Segrè, Universita degli Studi di Palermo, Palermo, Italy

^4^ – Instituto de Fısica Interdisciplinar y Sistemas Complejos IFISC (CSIC-UIB), Palma de Mallorca, Spain

^5^ – Department of Physics and Astronomy, University of Turku, Turku, Finland

^6^ – Complexity Science Hub, Vienna, Austria

**UIA EGO-NETWORK PLOTS AND TABLES**

In the main text, Table 1 describes the aggregated quantitative information about the links that UIA ICD code I67.1 has to other ICD codes and Figure 1 shows, as examples, two ego networks for female age cohorts of 40-49 and 50-59 years old. Below we show all the UIA ego networks for females and males. The nodes are colored uniformly throughout all the figures and the color-coding follows the letter coding within the ICD codes.

Figure S1: Ego network of I67.1 for female patients for age cohort 30-39.

Figure S2: Ego network of I67.1 for female patients for age cohort 40-49.

Figure S3: Ego network of I67.1 for female patients for age cohort 50-59.

Figure S4: Ego network of I67.1 for female patients for age cohort 60-69.

Figure S5: Ego network of I67.1 for female patients for age cohort 70-79.

Figure S6: Ego network of I67.1 for female patients for age cohort 80 or higher.

Figure S7: Ego network of I67.1 for male patients for age cohort 30-39.

Figure S8: Ego network of I67.1 for male patients for age cohort 40-49.

Figure S9: Ego network of I67.1 for male patients for age cohort 50-59.

Figure S10: Ego network of I67.1 for male patients for age cohort 60-69.

Figure S11: Ego network of I67.1 for male patients for age cohort 70-79.

**Table S1A:** First 34 ICD codes present in "ego networks" of ICD67.1 obtained from statistically validated networks of **women** for different age classes. For each row, the letter X indicates the presence of the ICD code in the "ego network" of the age class of column.


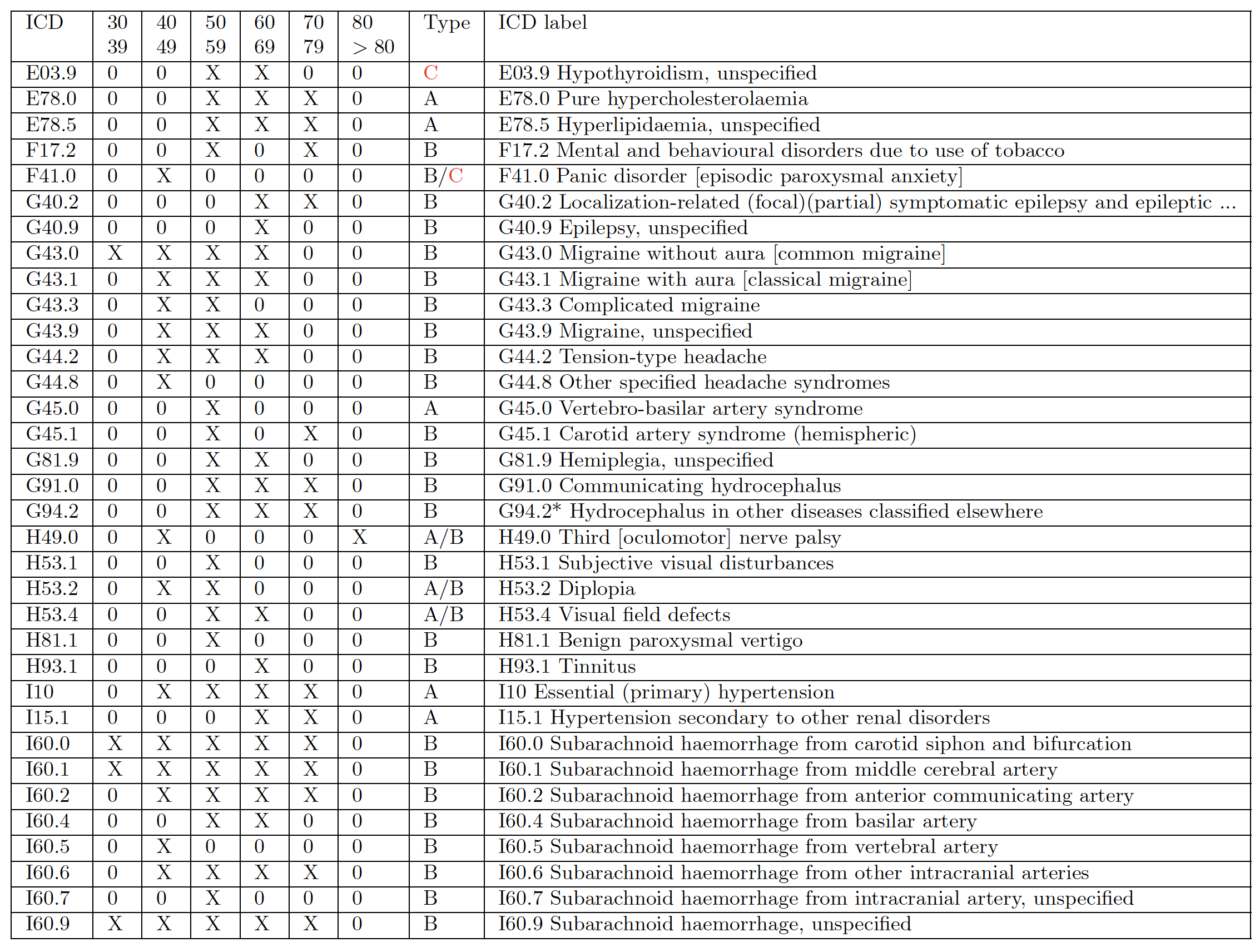


**Table S1B:** Remaining 39 ICD codes present in "ego networks" of ICD67.1 (included) obtained from statistically validated networks of **women** for different age classes. For each row, the letter X indicates the presence of the ICD code in the "ego network" of the age class of column.

**
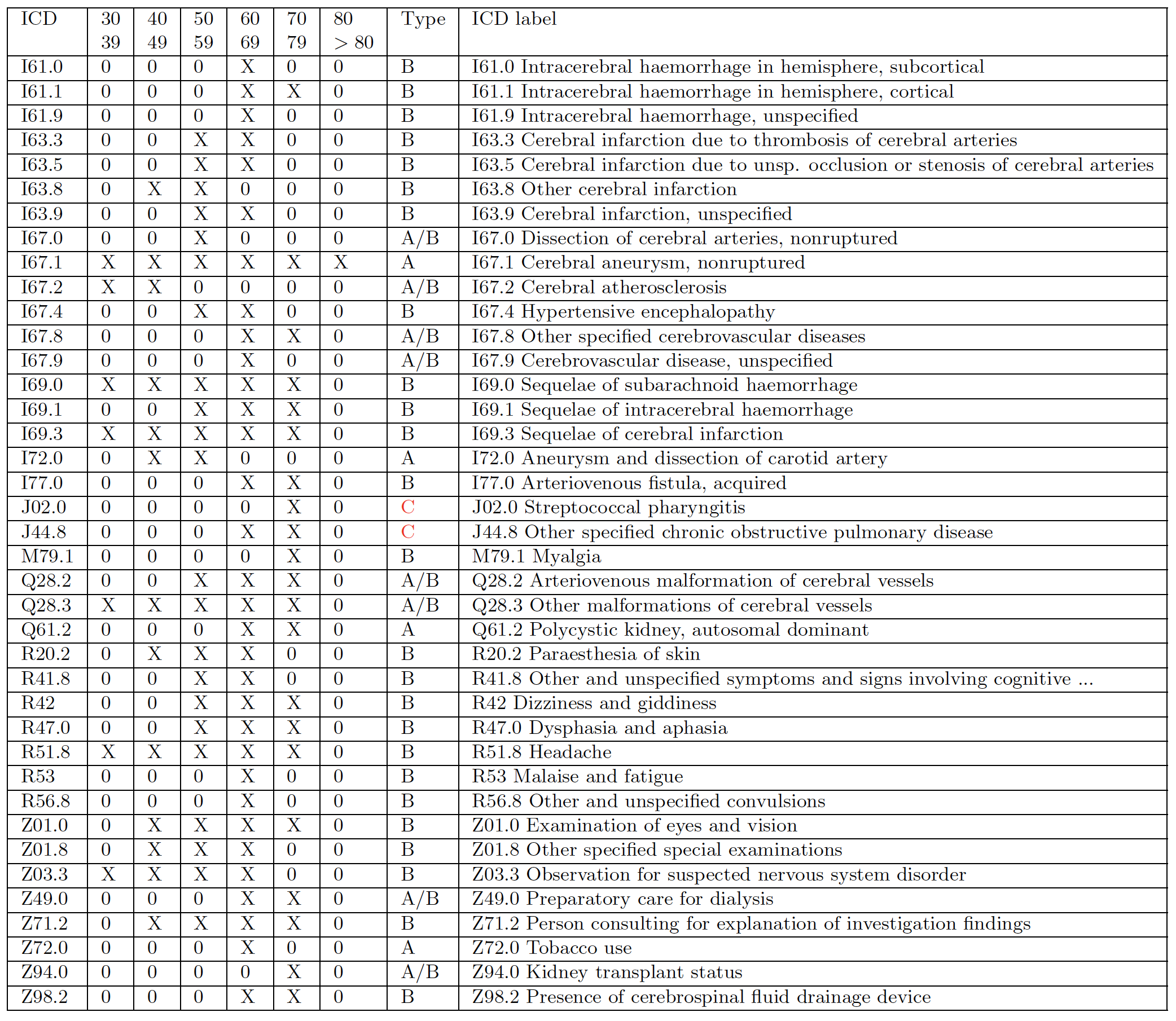
**

**Table S2:** 43 ICD codes present in "ego networks" of ICD67.1 (included) obtained from statistically validated networks of **men** for different age classes. For each row, the letter X indicates the presence of the ICD code in the "ego network" of the age class of each column.

**
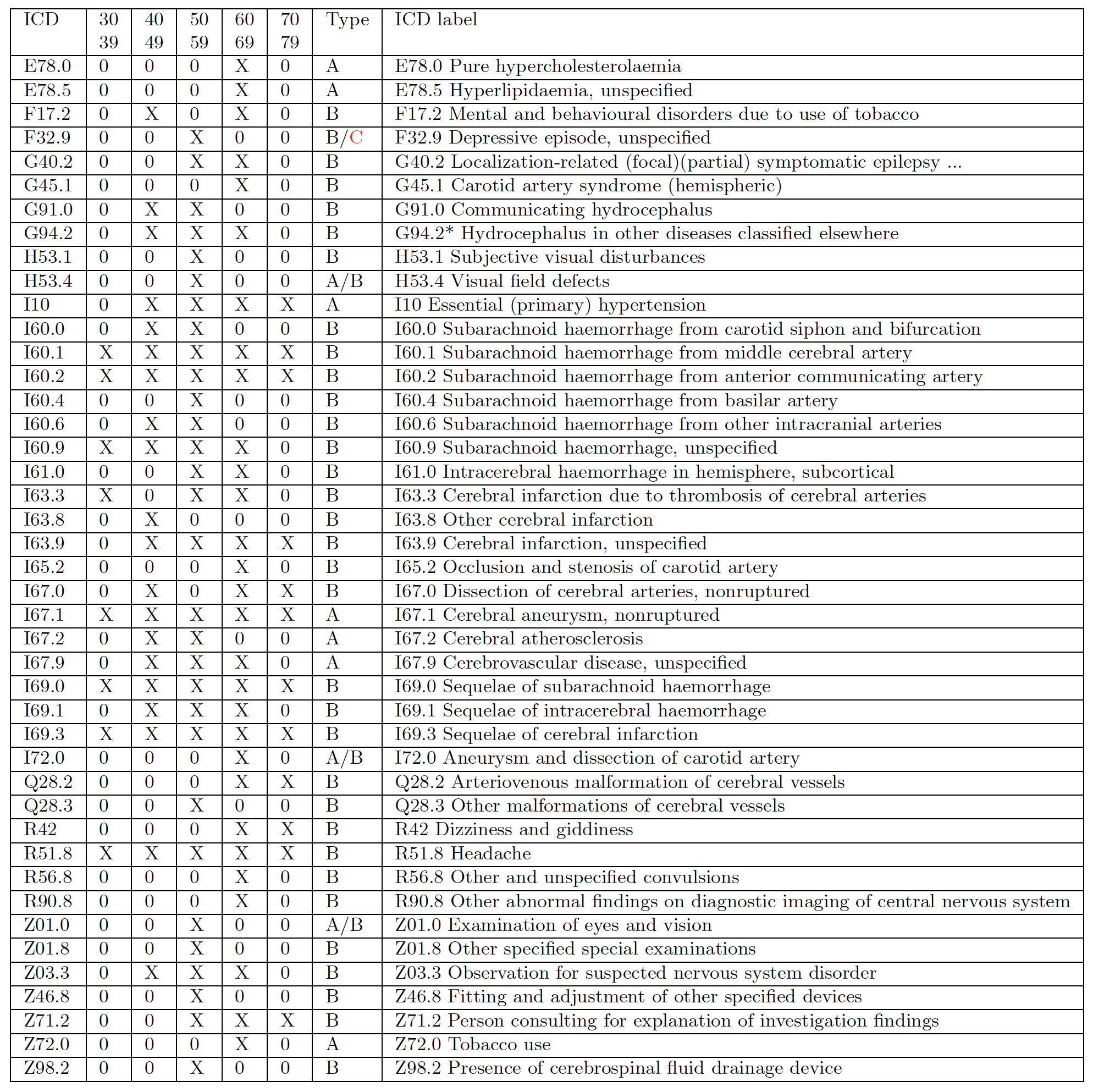
**
